# Charting the Unseen: How Non-Invasive Imaging Could Redefine Cardiovascular Prevention

**DOI:** 10.3390/jcdd11080245

**Published:** 2024-08-09

**Authors:** Giancarlo Trimarchi, Fausto Pizzino, Umberto Paradossi, Ignazio Alessio Gueli, Matteo Palazzini, Piero Gentile, Francesco Di Spigno, Enrico Ammirati, Andrea Garascia, Andrea Tedeschi, Daniela Aschieri

**Affiliations:** 1 Department of Clinical and Experimental Medicine, Cardiology Unit, University of Messina, 98124 Messina, Italy; giancarlo.trimarchi18@gmail.com; 2 Interdisciplinary Center for Health Sciences, Scuola Superiore Sant’Anna, 56127 Pisa, Italy; 3 Cardiology Unit, Heart Centre, Fondazione Gabriele Monasterio—Regione Toscana, 54100 Massa, Italy; fpizzino@ftgm.it (F.P.); uparadossi@ftgm.it (U.P.); igueli@ftgm.it (I.A.G.); 4 “De Gasperis” Cardio Center, Niguarda Hospital, ASST Grande Ospedale Metropolitano Niguarda, 20162 Milan, Italy; matteo.palazzini@ospedaleniguarda.it (M.P.); pierogentile.87@gmail.com (P.G.); enrico.ammirati@ospedaleniguarda.it (E.A.); andrea.garascia@ospedaleniguarda.it (A.G.); 5 Cardiology Unit of Emergency Department, Guglielmo da Saliceto Hospital, 29121 Piacenza, Italy; francesco.dispigno@yahoo.com (F.D.S.); d.aschieri@ausl.pc.it (D.A.)

**Keywords:** cardiovascular prevention, cardiovascular risk factors, echocardiography, cardiac resonance imaging, coronary computed tomography angiography

## Abstract

Cardiovascular diseases (CVDs) remain a major global health challenge, leading to significant morbidity and mortality while straining healthcare systems. Despite progress in medical treatments for CVDs, their increasing prevalence calls for a shift towards more effective prevention strategies. Traditional preventive approaches have centered around lifestyle changes, risk factors management, and medication. However, the integration of imaging methods offers a novel dimension in early disease detection, risk assessment, and ongoing monitoring of at-risk individuals. Imaging techniques such as supra-aortic trunks ultrasound, echocardiography, cardiac magnetic resonance, and coronary computed tomography angiography have broadened our understanding of the anatomical and functional aspects of cardiovascular health. These techniques enable personalized prevention strategies by providing detailed insights into the cardiac and vascular states, significantly enhancing our ability to combat the progression of CVDs. This review focuses on amalgamating current findings, technological innovations, and the impact of integrating advanced imaging modalities into cardiovascular risk prevention, aiming to offer a comprehensive perspective on their potential to transform preventive cardiology.

## 1. Introduction

Cardiovascular diseases (CVDs) continue to be a major global health concern, accounting for a significant portion of morbidity and mortality worldwide [1]. According to the World Health Organization (WHO), CVDs are responsible for approximately 17.9 million deaths each year, making them the leading cause of death globally [2]. The burden of CVDs extends beyond the individuals and families affected, placing substantial strain on healthcare systems and resources. CVDs encompass a range of conditions affecting the heart and blood vessels, including coronary artery disease (CAD), heart failure (HF), and stroke. These conditions often arise from underlying risk factors such as high blood pressure, high cholesterol, smoking, obesity, and physical inactivity. While advancements in medical science and therapeutic interventions have improved the outcomes for individuals with CVDs, the increasing prevalence of these conditions presents a growing challenge for healthcare providers and policymakers [3]. Traditionally, preventive measures have focused on lifestyle modifications, risk factor management, and pharmacological interventions to reduce the incidence of CVDs and their associated complications [4,5]. However, the role of imaging in cardiovascular prevention has gained increasing importance in recent years, offering a valuable tool for early detection, risk stratification, and the monitoring of individuals at risk for cardiovascular events [3,6]. 

Imaging modalities such as supra-aortic trunks ultrasound, echocardiography, computed tomography (CT) angiography, magnetic resonance imaging (MRI), and nuclear imaging techniques have revolutionized the field of cardiovascular prevention by providing detailed insights into the structure and function of the heart and blood vessels [7]. These imaging technologies enable healthcare providers to assess the presence of atherosclerosis, plaque burden, and other signs of cardiovascular pathology that may elevate the risk of future events such as heart attacks or strokes [8]. One of the key benefits of imaging in cardiovascular prevention is the ability to identify high-risk individuals before symptoms occur, allowing for early intervention and personalized treatment strategies [9]. For instance, imaging studies can identify individuals with subclinical atherosclerosis or cardiac dysfunction who might benefit from aggressive risk factor modification or preventive therapies to reduce their risk of future cardiovascular events [10]. In addition to early detection, imaging plays a pivotal role in risk stratification by providing valuable information on the severity and extent of CVDs. By quantifying the extent of atherosclerotic plaque, measuring cardiac function, and assessing the presence of CAD, imaging studies can help healthcare providers tailor treatment plans to each individual’s unique risk profile [11]. Furthermore, imaging technologies enable the ongoing monitoring of individuals at risk for CVDs, allowing for timely adjustments to treatment plans based on changes in disease progression or response to therapy. By incorporating imaging into routine cardiovascular assessments, healthcare providers can track disease progression, evaluate treatment effectiveness, and make informed decisions about the management of CVDs [3]. Advanced imaging modalities have opened new avenues for the early detection and characterization of CVDs, allowing for personalized preventive strategies. 

**Figure 1:**
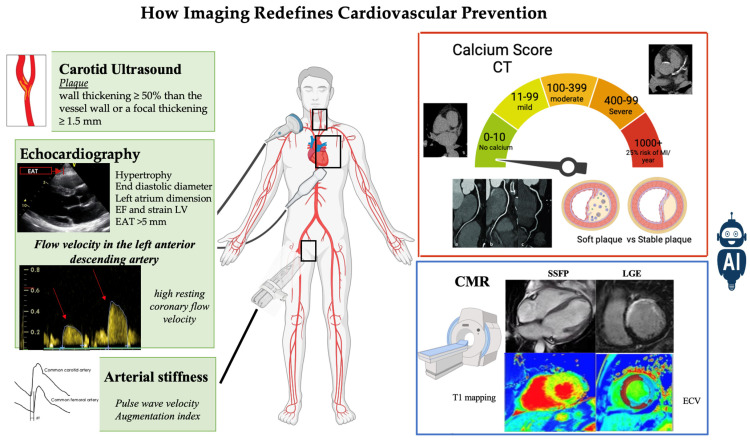
How imaging redefines cardiovascular prevention.

The primary objective of this narrative review is to provide a comprehensive overview of the role of advanced and emerging imaging techniques in cardiovascular risk assessment and the development of tailored preventive strategies, advocating for their widespread use in clinical practice. By thoroughly examining current evidence and technological breakthroughs in cardiovascular imaging, our goal is to provide an innovative perspective on how the use of these imaging techniques can redefine cardiovascular prevention strategies (Figure 1).

## 2. Natural History of Cardiovascular Disease: From Risk Factors to Clinical Manifestations

CVDs arise from a myriad of factors, including genetics, environment, and lifestyle choices. The natural history of CVDs is characterized by a gradual and intricate interplay of these elements, ultimately culminating in potentially life-threatening events if left unchecked. The journey of CVDs typically begins with unmodifiable risk factors such as genetics, age, and gender, which can predispose an individual to develop the condition [12]. These factors lay the foundation for potential cardiovascular issues, setting the stage for the influence of additional risk factors as time progresses. 

Among these latter, there must undoubtedly be mentioned diabetes, hypertension, dyslipidemia as well as environmental factors, such as diet, physical activity, and smoking, which also significantly contribute to the progression of CVDs [13]. Unhealthy behaviors, such as a poor diet and a sedentary lifestyle, can further amplify the risks associated with CVDs. Smoking, in particular, is a well-established risk factor for atherosclerosis [14,15]. As atherosclerosis advances, it may remain asymptomatic for years, progressing silently while simultaneously causing subclinical cardiac dysfunction. Adverse cardiac remodeling, extracellular matrix remodeling, and myocardial fibrosis are among the early signs of CVDs that often go unnoticed until they escalate into critical events [16]. In the absence of proper management and control of risk factors, a vulnerable plaque may rupture, leading to acute events such as acute myocardial infarction (AMI) and stroke, thereby exacerbating CVDs.

For those individuals already diagnosed with CVDs, whether clinically symptomatic or not, the control of risk factors and proper implementation of pharmacological and interventional therapies are essential for improving prognosis [17].

The natural history of CVDs is a multifaceted and dynamic process that involves a complex interplay of genetic, environmental, and lifestyle factors [18]. By understanding the intricate nature of CVDs and implementing appropriate interventions, individuals can mitigate their risk and improve their overall cardiovascular health. Ultimately, proactive management of risk factors and adherence to treatment regimens are crucial in managing the progression of CVDs and enhancing patient outcomes.

## 3. Cardiovascular Risk Stratification According to Current Guidelines

Screening for CVDs is of paramount importance given that atherosclerosis is a prolonged, progressive, and asymptomatic condition typically preceding the initial acute event in most individuals [19]. Considering that the initial clinical presentation of CVDs is AMI or sudden cardiac death (SCD) in 50% of cases, early detection of high-risk individuals plays a fundamental role in executing effective preventive measures within the community [20]. The guidelines from the European Society of Cardiology (ESC) [21] have recently been updated to advocate for the utilization of the Systematic COronary Risk Evaluation—2 (SCORE2) and SCORE2-Older Person (SCORE2-OP) risk assessment tools [22]. Concurrently, the American College of Cardiology (ACC) has revised the atherosclerotic cardiovascular disease (ASCVD) risk assessment score, collectively aiming to enhance risk evaluation within European and United States populations [23]. Specifically, the SCORE2 algorithm has been developed, calibrated, and validated to predict a 10-year risk of first-onset CVD in European populations, while SCORE2-OP has been similarly designed to assess the 10-year risk of cardiovascular events in individuals aged 65 years and older [22]. However, because risk factor scores rely on probabilistic calculations derived from population-based studies, these enhanced risk assessments do not universally apply to all individuals. Furthermore, these risk assessment tools exhibit certain limitations, which may account for recent findings indicating their failure to identify some high-risk patients [24]. While these scores represent a significant advancement in risk evaluation, they remain imperfect, particularly in certain subgroups such as individuals with a family history of premature CAD, those with chronic inflammatory diseases, and recreational drug users. The evaluation of the duration of exposure to risk factors also remains inadequate. Concerning non-modifiable risk factors, the scores tend to underestimate risk in younger individuals and women [25]. Emerging non-traditional risk factors and/or markers have been identified, but strategies for their detection, risk evaluation, and management are not well established, highlighting the necessity for further research in this domain. These markers include high-sensitivity C-reactive protein (hs-CRP), apolipoprotein B (ApoB), apolipoprotein A (ApoA), brain natriuretic peptides (BNP), troponin I, homocysteine, interleukins 1 and 6 (IL1, IL6), lipoprotein (a) [Lp(a)], cholesterol remnants, the size and number of low-density lipoprotein (LDL) particles, tissue/tumor necrosis factor-α (TNF-α), and uric acid [26]. 

All these factors and markers are linked with an increased risk of major adverse cardiovascular events (MACE), yet their exact role in risk assessment and the optimal strategies for addressing them requires clearer definitions. 

Cardiovascular imaging could play a crucial role in the assessment and management of ASCVD. The substrates of ASCVD, plaque characteristics, and progression, can be effectively monitored using various cardiovascular imaging techniques. This approach holds promise in ensuring that patients receive appropriate and timely treatment to prevent adverse cardiovascular events [27,28]. Despite its potential benefits, the current guidelines do not fully recognize the role of cardiovascular imaging in risk assessment for both primary and secondary prevention of ASCVD. As research continues to demonstrate the utility and efficacy of cardiovascular imaging in guiding clinical decision-making, it is essential for guidelines to evolve and incorporate this modern strategy into routine practice [29]. Ultimately, the integration of cardiovascular imaging into risk assessment algorithms might improve patient care and outcomes in the management of ASCVD.

## 4. Supra-Aortic Trunks Ultrasound in Cardiovascular Prevention

Echo-Doppler ultrasound of the supra-aortic trunks represents a non-invasive imaging modality that explores the main vessels originating from the aortic arch and extending to the cranial region and upper limbs. It offers real-time insights into the blood flow and identifies anomalies of the vessel wall [30,31].

A key feature of the Doppler ultrasound (DUS) is its ability to accurately measure the vessel wall and the residual lumen. This examination can detect the presence of plaques and provide information about their nature and the degree of stenosis [31]. A plaque is defined as a focal wall thickening ≥50% compared to the surrounding vessel wall or a focal thickening of ≥1.5 mm. As regards plaque characteristics, echolucency, inhomogeneity, and surface irregularity indicate unstable plaques prone to rupture, whereas smooth surfaces and normal or high echogenicity indicate more stable plaques [32]. For assessing carotid artery stenosis, the North American Symptomatic Carotid Endarterectomy Trial (NASCET) method is the most used. This method normalizes the smallest diameter or area of the stenotic tract to the diameter or area of the first plaque-free distal lumen [33]. An alternative method, derived from the European Carotid Surgery Trial (ECST), measures the reference diameter or area at the same point of the stenosis [34]. The NASCET is preferred because it correlates better with hemodynamics and angiography [35], while the ECST method better correlates with the atherosclerotic burden. The 2017 ESC Guidelines on the Diagnosis and Treatment of Peripheral Arterial Diseases recommend the use of the NASCET method for the estimation of carotid stenosis [36,37]. The accurate assessment of the degree of stenosis is crucial in managing patients, as the ESC guidelines recommend revascularization for asymptomatic patients with 60–99% stenosis and symptomatic patients with 50–99% stenosis. Occluded vessels are not candidates for invasive treatment; however, identifying them is important because they represent a risk modifier for contralateral stenosis [36]. 

Another value derived from DUS analysis of the arterial wall is the intima-media thickness (IMT), which measures the thickness of the intima and media layers. These two components of the vessel wall can be easily distinguished by DUS, as the intima is the highly echogenic internal part of the wall, and the media is a less echogenic layer just external to the intima (double-line pattern). The measurement should be taken on a 10 mm plaque-free segment of the far carotid wall, at least 5 mm below the distal end of the common carotid artery. It is generally recommended to perform the measurement using a semi-automated system [38]. Normal values of IMT are not well established. However, the latest ESH/ESC hypertension guidelines [39] reconfirm that a carotid IMT > 0.9 mm is a marker of asymptomatic organ damage, although, in middle-aged and elderly patients, higher threshold values indicate high cardiovascular risk [32,40]. Numerous studies have demonstrated that carotid DUS assessment, including IMT and the detection of carotid plaques, can significantly predict atherosclerosis and CAD independently and incrementally to Framingham risk score and across all pre-test risk probability categories [41,42]. Nevertheless, its systematic use to improve CVD risk assessment is not recommended due to the lack of methodological standardization [41].

Beyond atherosclerotic disease, DUS can also detect other pathologies such as dissection, intramural hematoma, and fibromuscular dysplasia [43,44]. 

**Figure 2:**
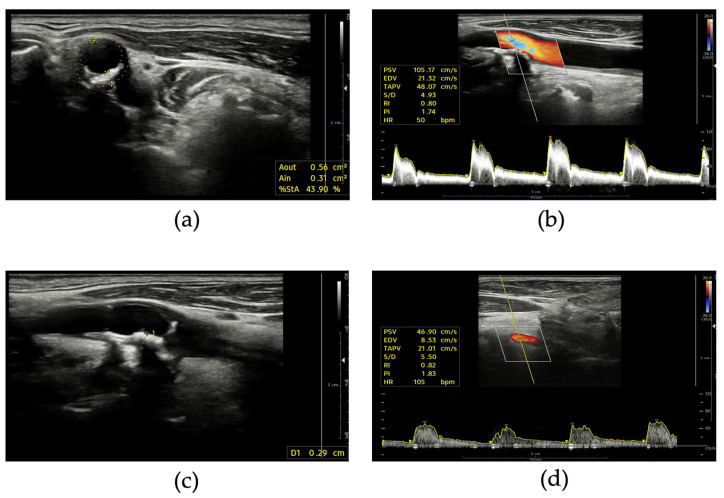
Supra-aortic trunks ultrasound: (**a**) short axis view of internal carotid artery stenosis with calcific plaque; (**b**) pulsed wave Doppler spectrum of an internal carotid artery with not hemodynamically relevant stenosis; (**c**) long axis view of a calcific plaque of internal carotid artery; (**d**) normal pulsed wave Doppler spectrum of a vertebral artery.

The measurement of flow velocities and the estimation of the hemodynamic burden of stenosis is the real incremental value of DUS in comparison to angiography or computed tomography (CT) (Figure 2). It is important to measure both intra-stenotic peak velocity and post-stenotic velocity. Different thresholds have been proposed for peak velocity: typically, values of >125–160 cm/s identify stenosis ≥50% according to the NASCET method, while peak velocities of 200–230 cm/s are considered consistent with stenosis ≥70% [34,45]. A post-stenotic velocity < 50 cm/s indicates significant stenosis. The internal carotid artery/common carotid artery peak systolic velocity ratio is also a useful parameter with values ≥ 2 in identifying stenosis ≥50% [46].

The investigation of subclavian arteries is also important, as it can reveal plaques or stenosis associated with subclavian stenosis syndrome, upper limb claudication, and coronary steal, in the case of coronary artery bypass grafting [47]. 

In conclusion, DUS is a fundamental first-line technique for the early detection of vascular disease. Its availability, repeatability, and low cost are the main advantages making DUS the most widely used method in this setting. Moreover, it can contribute to risk stratification and help inform tailored preventive measures and revascularization. According to the 2021 ESC Guidelines on Cardiovascular Prevention, this technique may be considered as a risk modifier in patients at intermediate risk when a Calcium Artery Coronary (CAC) score is not feasible [21].

## 5. Arterial Stiffness and Pulse Wave Velocity 

Arterial stiffness refers to the reduced ability of the arteries to expand and contract in response to changes in blood pressure. This stiffness is often caused by the hardening and thickening of the arterial walls due to factors such as aging, high blood pressure, smoking, diabetes, and high cholesterol levels. Increased arterial stiffness is a significant predictor of CVDs and all-cause mortality, making it an important aspect of cardiovascular health to monitor and manage [48,49,50]. One common method for measuring arterial stiffness is pulse wave velocity (PWV). PWV is determined by recording the arterial pulse wave at a proximal artery, typically the common carotid, and a distal vessel, such as the femoral artery. These arteries are selected because of their superficial location and the fact that the distance between them approximates the aortic measurement [51,52]. A pulse tonometer is used to measure PWV, which expresses the time necessary for the pulse wave to travel between the two points. A higher PWV correlates with greater arterial wall stiffness [53].

Another indirect method of measuring arterial stiffness is the arterial augmentation index, which is calculated as the augmentation pressure divided by the pulse pressure multiplied by 100 [54]. Increased arterial stiffness results in faster propagation of the forward pulse wave and a more rapid reflected wave, leading to a higher augmentation index, indicating higher arterial stiffness [54].

While PWV and the augmentation index have not been included in recent ESC Guidelines on CVD prevention [21], studies have shown their correlation with the risk of developing CVD and all-cause mortality [55,56]. A large meta-analysis from 2014 demonstrated that PWV was an independent predictor of CAD, stroke, and cardiovascular events. Moreover, PWV has been shown to improve risk prediction in certain subgroups, such as those with intermediate CVD risk [57].

Arterial stiffness is also linked to other cardiovascular conditions, such as impaired diastolic function [58], and altered coronary blood flow [59]. Studies have even shown that arterial stiffness can be present in hypercholesterolemic children, suggesting a potential role in the early development of atherosclerosis [60,61]. Overall, arterial stiffness is a significant factor in cardiovascular health and can be used to measure vascular aging and estimate cardiovascular risk [50,62].

**Figure 3:**
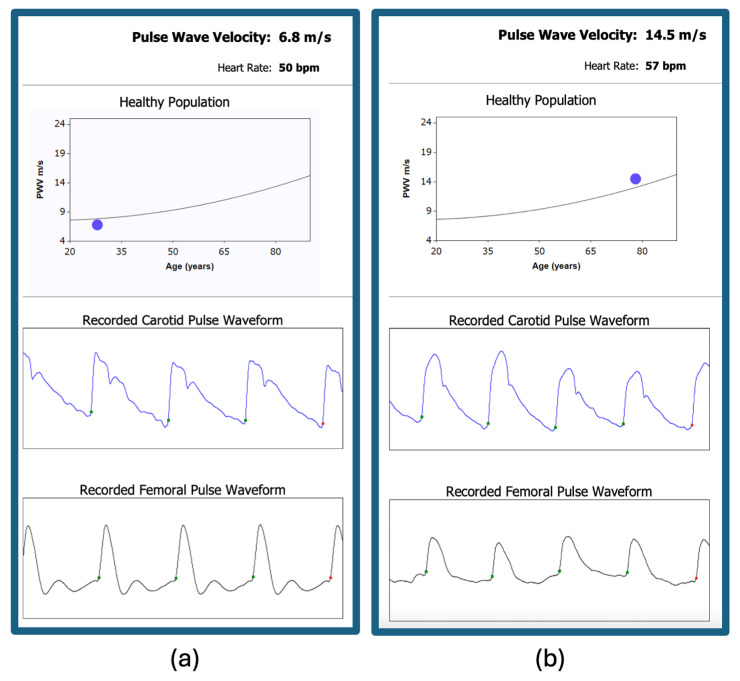
Carotid-femoral pulse wave velocity measurement: (**a**) normal value of pulse wave velocity in a young man without cardiovascular risk factors; (**b**) increased pulse wave velocity in a 78-year-old male smoker with hypertension and type 2 diabetes.

Despite the benefits of measuring arterial stiffness, there are challenges associated with these methods, including the difficulty of measuring and the lack of reproducibility [63]. These factors have limited the widespread use of PWV and the augmentation index in clinical practice. However, research continues to support the importance of assessing arterial stiffness for predicting cardiovascular risk and guiding treatment decisions. As our understanding of arterial stiffness grows, these methods may become more integrated into routine cardiovascular assessments, providing valuable information for improving patient outcomes. Figure 3 illustrates a clinical scenario for applying PWV measurement.

## 6. The Role of Echocardiography in Cardiovascular Risk Assessment

Echocardiography is a widely used imaging technique in the field of cardiology, providing valuable information about the heart’s structure and function [9]. While there is limited evidence that echocardiography improves CVD risk assessment in primary prevention, its potential role in detecting subtle anomalies before their clinical manifestation could be highly valuable on an individual basis. The role of traditional echocardiographic markers, such as wall hypertrophy, diastolic dysfunction, and left atrial and aortic root dilation, in tailored risk stratification, is well established. Additionally, novel markers like longitudinal strain, 3D morphological and functional evaluation, and epicardial adipose tissue (EAT) are emerging as potential tools for more precise risk stratification and guide personalized preventive strategies [64].

A recent meta-analysis associated these markers with an increased risk of adverse events in individuals without known CVD [64]. Additionally, echocardiography plays a crucial role in evaluating left ventricular (LV) systolic and diastolic functions, both through traditional and more recent methods [65]. It has demonstrated incremental value in predicting the evolution of HF and cardiovascular events in patients with asymptomatic HF [66,67]. In young athletes, echocardiography is essential for screening and early identification of structural heart diseases, which could lead to SCD in this population [68].

Furthermore, in asymptomatic patients with cardiovascular risk factors and normal LV function, tissue Doppler imaging has been correlated with additional prognostic value, suggesting its potential role in tailored preventive treatment [69]. Speckle tracking echocardiography and strain imaging of the LV are gaining importance in assessing ventricular function and for predicting mortality better than LV ejection fraction (LVEF) and the wall motion score index [66], particularly in cardiomyopathies [70,71,72]. In cardio-oncology, LV strain is critical for detecting and evaluating drug toxicity, even when LVEF is normal or mildly reduced [73].

Another emerging aspect of echocardiography is the evaluation of EAT. A larger amount of EAT revealed during echocardiographic evaluation has been correlated with the risk of atherosclerosis and its severity [74,75]. Notably, EAT may potentially be a therapeutic target for novel cardiometabolic medications that modulate adipose tissue. Its ease of detection makes it an important factor in cardiovascular prevention and treatment, complementing other cardiovascular imaging methods, including deep learning technologies [76,77]. 

The evaluation of EAT through echocardiography has certain limitations due to limited agreement between junior and senior observers when measuring echocardiographic EAT [78]. Additionally, there is a modest correlation between EAT and epicardial fat volume measured by CMR. However, despite these limitations, measuring echocardiographic EAT thickness may still be valuable as a research tool in larger datasets to investigate associations with cardiovascular disease [79,80,81].

Transthoracic echocardiography also allows for the measurement of coronary flow velocity in the left anterior descending artery, providing valuable information on the coronary flow reserve. An elevated resting coronary flow velocity has been linked to a reduced coronary flow velocity reserve and worse long-term prognosis in patients with chronic coronary syndrome [82]. In patients with ischemic and non-ischemic HF, a high resting coronary flow velocity has been associated with worse survival outcomes, independently and in addition to other cardiac parameters [82].

In conclusion, while echocardiography may not be routinely recommended in primary CVD prevention and risk assessment, its potential for individual risk stratification and personalized preventive strategies should not be overlooked. Emerging echocardiographic markers and techniques, such as strain imaging, EAT evaluation, and assessment of coronary flow velocity, have shown promising results in improving risk assessment and guiding treatment strategies across various cardiovascular conditions. Further research and larger studies are needed to establish the clinical utility of these novel echocardiographic markers and techniques in cardiovascular risk assessment and management.

## 7. Coronary Artery Calcium Score

Estimating the risk of adverse cardiovascular events using clinical calculators can be particularly challenging for patients with risk scores near the decision threshold. Therefore, it is crucial to identify tools that can optimize risk stratification, especially for those with low to moderate cardiovascular risk [21]. 

**Figure 4:**
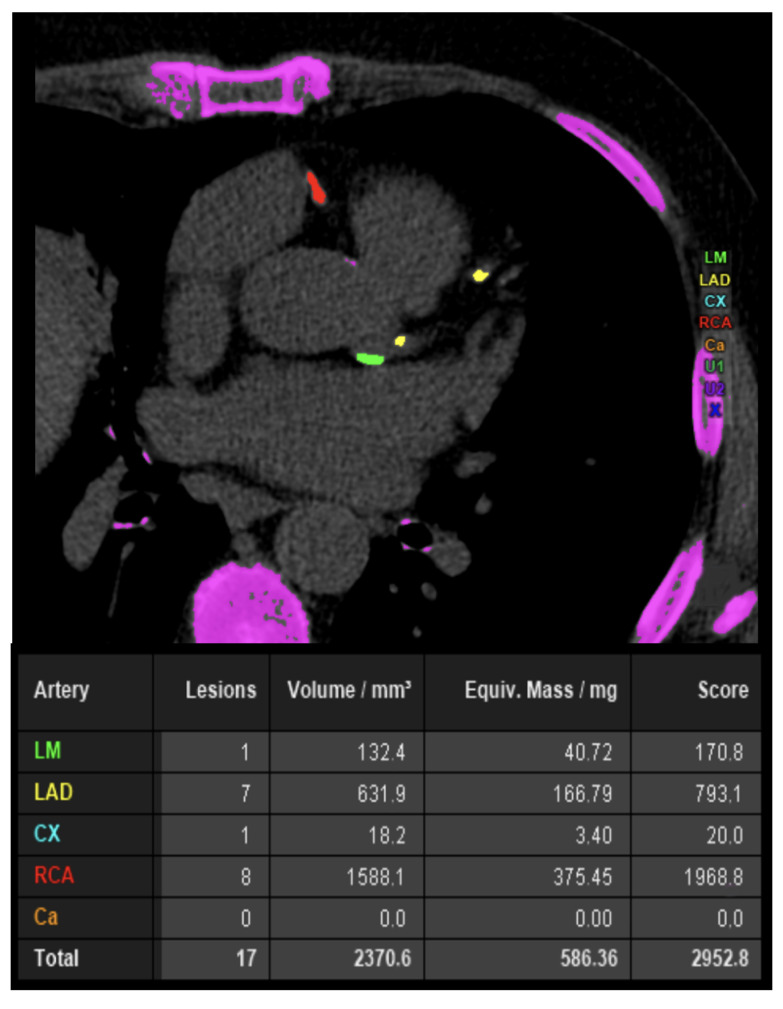
A 65-year-old male smoker with hypertension and dyslipidemia underwent coronary artery calcium (CAC) score calculation for exertional angina. CAC score value is increased in the absence of critic lesions. CAC—coronary artery calcium; CCTA—coronary computed tomography angiography; Cx—circumflex artery; LAD—left anterior descending artery; LM—left main.

The CAC score is a continuous measure, whose determination is performed through axial scans of cardiac CT, without the use of contrast (Figure 4). The effective dose of radiation is usually low, comparable to that of a mammogram [83]. Calcification within the epicardial coronary arteries is identified as an area of hyper-attenuation ≥1 mm^2^ or ≥3 adjacent pixels with >130 Hounsfield units (HU) [83]. The Agatston method is the commonly used quantification system providing age- and gender-specific thresholds. CAC score enhances risk discrimination and classification beyond the Framingham risk score, regardless of risk strata [84], enabling the prescription of preventive therapies for effective primary prevention [85,86]. This diagnostic test’s potential is recognized by the latest ESC and ACC/ American Heart Association (AHA) guidelines on CVD prevention, which consider CAC a “risk modifier”, for reclassifying risk upward or downward [21,23]. 

However, CAC has many limitations. While individuals with a high calcium burden exhibit a higher rate of cardiovascular events, nevertheless, in the Multi-Ethnic Study of Atherosclerosis (MESA) cohort, 32% of patients with a CAC score of zero experienced a cardiovascular adverse event over a median follow-up of 8.5 years [87,88]. There is a disconnection between zero calcium and zero atherosclerosis: calcified plaque represents a small proportion of the atherosclerotic burden. Calcifications occur in a later stage of the coronary artery plaque continuum, suggesting that the true plaque burden might be underestimated by the CAC score. Therefore, while a zero CAC score is more reassuring in older patients, indicating an absence of significant plaque burden, deferring preventive therapies in young patients based on a zero CAC score can be risky, considering their lifetime risk and the long-term benefits of pharmacologic prevention [88]. 

Coronary calcium is an integral part of the atherosclerotic process. Knowing the CAC score can guide clinical decisions, by providing insights into a patient’s risk stratification beyond what clinical assessment alone can offer.

## 8. Coronary Computed Tomography Angiography 

**Figure 5:**
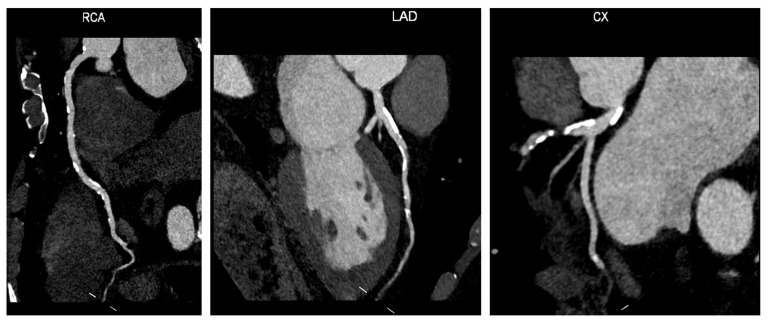
Coronary computed tomography angiography: A 75-year-old female patient with hypertension, diabetes, and dyslipidemia underwent CCTA as a screening procedure before TAVI intervention. The CCTA shows widespread atherosclerotic disease in all three vessels with extensive non-critical calcified lesions. Abbreviations: CCTA—coronary computed tomography angiography; Cx—circumflex artery; LAD—left anterior descending artery; RCA—right coronary artery; TAVI—transcatheter aortic valve implantation.

Coronary CT angiography (CCTA) is a powerful tool for identifying coronary stenoses and predicting cardiac events in patients with suspected or known CAD (Figure 5) [89,90]. Numerous studies have shown that CCTA can significantly enhance prognostic assessment, leading to improved risk prediction and potentially reducing adverse cardiovascular events such as AMI or coronary death [91,92]. 

Several key studies have highlighted the benefits of CCTA in assessing CAD. For instance, a 2009 study involving 1256 patients with suspected CAD demonstrated that CCTA had a significant prognostic impact on predicting cardiac events for over one year [93]. This study also identified a patient population with a lower event risk than that predicted by conventional risk factors alone, underscoring the added value of CCTA in risk prediction [94]. 

The CONFIRM (COronary CT Angiography EvaluatioN For Clinical Outcomes: An InteRnational Multicenter Registry) registry, a large-scale study conducted across multiple centers and countries, further validated the prognostic value of CCTA. In asymptomatic individuals, CCTA was found to stratify prognosis, though its additional risk-predictive advantage was not deemed clinically significant compared to a classic risk model based on the CAC score [95]. However, in individuals with angina and suspected CAD, CCTA was shown to offer incremental discriminatory power over CAC for evaluating those at risk of adverse cardiovascular events [96]. 

While CCTA has been studied in various CVD prevention settings, there is limited evidence supporting its use in this context, particularly in comparison to the CAC score [97]. Nonetheless, recent trials such as SCOT-HEART (Scottish Computed Tomography of the Heart) and ISCHEMIA (International Study of Comparative Health Effectiveness with Medical and Invasive Approaches) have demonstrated that the severity of atherosclerotic disease defined by CCTA correlates with the risk of adverse cardiovascular events [91,98]. In addition to identifying obstructive stenosis, CCTA plays a crucial role in evaluating non-obstructive CAD, which can also contribute to adverse cardiovascular outcomes. Recent advancements in CCTA imaging techniques have enabled the quantification and characterization of atherosclerotic plaques, by assessing the extent of CAD and differentiating between various plaque features [99]. This includes evaluating plaque composition, location, vulnerability, and other morphologic and functional features that can impact clinical outcomes [100]. The ability to evaluate atherosclerotic plaques and perform prognostic stratification through CCTA has also been supported by a sub-study of the COURAGE (Clinical Outcomes Utilizing Revascularization and Aggressive Drug Evaluation) trial [101]. 

In this context, CCTA enables the thorough assessment of specific plaque characteristics that classify the plaque as “high-risk” (HRP) for future events. These characteristics include low attenuation plaque (LAP), spotty or micro-calcifications, positive remodeling, and the napkin ring sign (NRS). Among these features, NRS is the only operator-dependent one. A recent expert consensus has suggested the use of different Hounsfield units (HU) cut-offs to identify LAP [102]. Specifically, HU > 350 indicates dense calcium, 131 < HU < 150 indicates a fibrous plaque, 31 < HU < 130 indicates a fibro-fatty plaque, and −30 < HU < 31 indicates a necrotic core, with LAP being indicated by the category of HU < 30 [102]. While some researchers use HU < 60 to identify LAP [103], larger trials like SCOT-HEART have included HU < 30 in their criteria [104], and the Coronary Artery Disease Reporting and Data System (CAD-RADS) 2.0 also defines LAP as HU < 30 in its reporting algorithm [105]. Positive remodeling is an early compensatory mechanism in atherosclerosis, characterized by vessel wall and lumen enlargement to delay lumen obstruction, and is associated with extended lipid necrotic core, serving as a marker of plaque instability [106,107,108]. In CCTA, positive remodeling can be recognized by outward plaque growth, the presence of atherosclerosis, minimal lumen loss, or a ratio of the outer vessel diameter at the plaque site divided by the average outer diameter of the proximal and distal vessels being greater than 1.1 [105].

The presence of spotty calcification or microcalcification in atherosclerotic plaques is considered a high-risk feature associated with thin fibrous cap fibroatheroma (TCFA) and increased lipid index, while the number of calcifications may be linked to decreased fibrous cap thickness [109,110]. Spotty calcifications are visualized in CCTA as punctate calcium within a plaque (or calcium < 3 mm) [105]. Necrotic core with a surrounding hyper-attenuated ring-like area, known as NRS, is a marker of vulnerable plaques [111]. 

NRS is recognized in CCTA as a central low-attenuation plaque area in contact with the lumen and a hyperattenuated rim around it [99]. It is important to note that NRS is the sole qualitative finding in assessing high-risk plaques by CCTA. In the CAD-RADS 2.0 score, the presence of two or more high-risk features is indicated in the score result. The updated CAD-RADS reporting system for CCTA suggests including identifications of such characteristics as an “HRP indication” in the report following the CAD-RADS score [105]. 

Further establishing these criteria, The Society of Cardiovascular Computed Tomography and the North American Society of Cardiovascular Imaging (SCCT/NASCI) published an expert consensus in 2020 on assessing atherosclerotic plaque characteristics in CT scans, discussing the association of these characteristics with plaque vulnerability and outcomes and updating previous guidelines [112]. The consensus also provides guidance on technical considerations, uniform reporting, and implications for vulnerable plaque treatment [102]. Key studies demonstrating the prognostic value of high-risk CCTA plaque characteristics are listed in Table 1.

## 9. Standard and AI-Based Computer Tomography-Derived Flow Fractional Reserve

The evaluation of hemodynamically significant stenosis has been greatly enhanced by recent advancements in CT software applications. A new noninvasive technology known as coronary computed tomography fractional flow reserve (CT-FFR) has been developed, which utilizes CTA images along with computational fluid dynamic (CFD) techniques to calculate virtual FFR values in coronary vessels. This technology provides a more precise quantification of specific coronary lesions and their hemodynamic impact [120,121].

A three-dimensional (3D) anatomical model of the coronary arteries is generated for the specific patient through the application of semiautomatic contouring and segmentation techniques [122]. Subsequently, a physiological model is developed, incorporating patient-specific hemodynamic conditions for inflow and outflow, with resting myocardial blood flow proportionate to the myocardial mass. In this model, microvascular resistance is inversely related to the size of the epicardial coronary arteries. Furthermore, the hyperemic reduction in microvascular resistance induced by adenosine is also simulated, thereby eliminating the requirement for adenosine infusion [122].

Performing CT-FFR proves to be highly beneficial in cases of intermediate-risk anatomy, which is characterized by the presence of one or two intermediate stenotic lesions. These lesions refer to a luminal stenosis reduction ranging from 30% to 69%, or vessels with stenosis greater than or equal to 70% apart from the left main or LAD arteries. CT-FFR is particularly valuable in the intermediate-risk group for determining subsequent management decisions, such as choosing between optimal medical therapy (OMT) and invasive coronary angiography (ICA) with revascularization. When CT-FFR results show normal values (>0.8) in this category, patients can effectively undergo treatment with OMT [123,124]. In such cases, ICA and revascularization procedures can be safely delayed, leading to lower incidences of myocardial infarction, major adverse cardiovascular events (MACE), cardiac death, and revascularization beyond 90 days [124].

The Synergy Between Percutaneous Coronary Intervention With Taxus and Cardiac Surgery III Revolution) (SYNTAX III Revolution) trial involving two heart teams demonstrated that utilizing both CTA and CT-FFR resulted in altering the treatment decision for 7% of patients, changing the identified vessel for revascularization in 12%, and reclassifying 14–16% of patients as having a lower SYNTAX score [125]. The Does Routine Pressure Wire Assessment Influence Management Strategy at Coronary Angiography for Diagnosis of Chest Pain (RIPCORD) study, which included three interventional cardiologists, revealed that CT-FFR influenced the management strategy (medical treatment versus PCI versus coronary artery bypass graft) for 36% of patients when compared to CTA alone. This ultimately led to a 30% decrease in PCI procedures and a shift in the chosen vessel for PCI for 18% of patients [126].

However, CFD-based CT-FFR presents potential limitations, such as off-site computation and lengthy processing times. Accordingly, new technical approaches have emerged to address these limitations, particularly those involving software algorithms that leverage artificial intelligence (AI) and machine learning (ML) based on deep neural networks [127,128]. Recent studies have demonstrated that ML-based CT-FFR (CT-FFRML) mitigates these drawbacks inherent to CFD-based CT-FFR, while also offering a higher prognostic value compared to assessing flow-limiting CAD through CCTA alone. This advancement enhances the efficiency of ICA and minimizes associated complications [129,130,131].

Several single-center studies have provided valuable insights into the diagnostic performance of CT-FFRML. For example, Tesche et al. conducted a retrospective study analyzing 85 patients (104 vessels), which showed sensitivities of 79% and 90% and specificities of 94% and 95% on a per-lesion and per-patient level, respectively [132]. The study also revealed the superior diagnostic performance of CT-FFRML compared to CCTA alone, with higher AUCs of 0.89 vs. 0.61 and 0.91 vs. 0.65 on both levels [132]. Another publication by a different group of researchers further supported the efficacy of CT-FFRML in detecting lesion-specific ischemia when combined with plaque quantification in 84 patients (103 vessels) [130]. This study showed a significantly better discrimination ability compared to CCTA, with an AUC of 0.89 vs. 0.61, along with sensitivities and specificities of 82% and 94% [130]. Similarly, Rother et al. demonstrated comparable results with a sensitivity and specificity of 91% and 96% in a study involving 71 patients (91 vessels) [133]. Their study also highlighted the significantly higher AUC for CT-FFRML compared to visual stenosis grading on CCTA, with AUCs of 0.94 vs. 0.61 [133]. These findings collectively suggest the clinical benefits of utilizing CT-FFRML for accurate diagnosis and management of coronary artery disease.

A collaborative effort among multiple centers was established based on the MACHINE registry, (known as Diagnostic Accuracy of a Machine-Learning Approach to Coronary Computed Tomographic Angiography-Based Fractional Flow Reserve: Results from the MACHINE Consortium) [129]. This trial involved 351 patients (525 vessels) from five different sites across the United States, Europe, and Asia. The main goal of the study was to assess the diagnostic performance of CT-FFRML in comparison to CT-FFR, utilizing a CFD algorithm (CT-FFRCFD). The study used first- and second-generation dual-source CT scanners from Siemens Healthineers and a research prototype (cFFR version 2.1) for CT-FFRML analysis [129]. Results from the trial showed a significant enhancement in diagnostic accuracy with CT-FFRML compared to CCTA (78% vs. 58%). Additionally, the accuracy per patient improved from 71% with CCTA to 85% with machine learning-based CT-FFR, with a correction of 73% false-positive CCTA results. It was suggested that CT-FFRML has the potential to differentiate between the need for ICA with revascularization and conservative therapy [129]. Tang et al. conducted an investigation involving 136 patients (122 vessels) with intermediate coronary lesion severity, demonstrating high accuracy and specificity using CT-FFRML for identifying lesion-specific ischemia compared to CCTA grading alone [134]. Similarly, Nous et al. presented comparable results, showing a significant decrease in the number of patients requiring additional ICA with the availability of on-site CT-FFR alongside CCTA [135], significantly changing the initial management strategy. 

Finally, another application of CFD in CCTA involves wall shear stress (WSS), which represents the tangential frictional force of blood on the coronary vessel wall [136]. Vascular biology has long associated WSS with coronary atherosclerosis through changes in endothelial cell pathways. Notably, low shear stress is linked to vascular cell adhesion molecule (VCAM), crucial in atherosclerosis pathogenesis [137,138,139,140].

WSS derived from CCTA aids in identifying high-risk plaque beyond percent stenosis and is independently related to an increased coronary plaque burden [141,142]. The EMERALD study [143] showed that integrating hemodynamic indices like WSS from CCTA with FFRCT, changes in FFRCT, and axial plaque stress provided incremental prognostic value. This was above anatomic stenosis severity and CCTA-derived plaque traits, indicating that WSS assessment could help identify lesions leading to ACS and plaques with high-risk features. Therefore, considering WSS in CCTA analysis could identify at-risk patients and guide management strategies for CAD patients.

## 10. Photon-Counting CT and Its Applications in Cardiovascular Diagnostics

The widespread acceptance of cardiovascular CT can be credited to its non-invasive nature and its capacity to produce high-quality images with rapid acquisition times and three-dimensional reconstructions. However, there remains a critical requirement to enhance contrast resolution and image quality while minimizing radiation exposure, diminishing blooming or beam-hardening artifacts, and augmenting tissue characterization capabilities [144,145]. Photon counting CT (PCCT) emerges as a promising technology with the potential to enhance cardiovascular CT imaging and achieve these desired objectives [146]. PCCT employs photon-counting detectors (PCDs), which offer numerous advantages over conventional energy-integrating detectors (EIDs) utilized in traditional CT scanners, such as superior spatial resolution, enhanced contrast, and reductions in noise and artifacts [147].

Several studies have demonstrated the benefits of PCCT in enhancing measurements of plaque volume and stenosis severity. These benefits are largely due to improved spatial resolution, better soft-tissue contrast, and reduced noise. Si-Mohamed et al. [148] found in a phantom study that PCCT images had a detectability index that was 2.3 times higher for coronary lumen and 2.9 times higher for non-calcified plaque compared to EID-CT images. Clinical validation in 14 patients showed that PCCT offered superior image quality and diagnostic confidence, as assessed by three radiologists: This improvement was primarily attributed to a significant reduction in blooming artifacts compared to EID-CT [148].

PCCT’s capabilities are particularly beneficial for evaluating luminal stenosis in heavily calcified plaques. Koons et al. simulated coronary arteries with various calcifications and found that PCCT, when compared to conventional CT at the same dose, offered better visualization of calcium plaques and patent lumen, and greater accuracy in quantifying luminal stenosis across all plaque types [149]. Additionally, Allmendinger et al. [150] tested a new calcium-removal image reconstruction algorithm named PureLumen. Their study focused on removing calcified contributions in an anthropomorphic thorax phantom. The algorithm successfully reduced blooming artifacts, leading to clearer and more precise imaging [150].

The advantages of PCCT are evident in CAC assessment. A phantom study showed that PCCT, compared to conventional CT, enhanced CAC detectability and provided more precise volume scores at a reduced slice thickness [151]. The study found that using monoenergetic reconstructions at 70 keV for PCCT at different tube potentials allowed reproducible Agatston scores for medium- and high-density CAC. This indicates that PCCT offers consistent and accurate CAC assessment using specific settings [151]. 

An ex vivo study on cadaveric hearts demonstrated excellent correlation and agreement between Agatston scores from conventional CT and PCCT, suggesting a potential for converting the established Agatston score from EID-CT to PCCT. The study also showed good inter-scan reproducibility for both PCD-CT and EID-CT [152].

Eberhard et al. found that PCCT accurately quantified the CAC burden. They noted that more accurate and reliable CAC scoring could be achieved by optimizing the iterative reconstruction algorithm and increasing the keV levels of virtual monoenergetic images [153].

PCCT has potential in plaque assessment by overcoming conventional CT limitations and better distinguishing between stable and unstable atherosclerosis. Mergen et al. scanned 20 patients with atherosclerotic plaques in the proximal coronary arteries using PCCT, emphasizing the ultra-high-resolution mode’s ability to reduce blooming artifacts and enhance the depiction of fibrotic and lipid-rich plaque components [154]. 

In an in vitro study, Rotzinger et al. simulated different patient sizes (small, medium, and large) and demonstrated PCCT’s superiority over conventional EID-CT in detecting simulated non-calcified and lipid-rich plaques in coronary arteries [155]. 

In a study by Boussel et al., 23 plaques (10 calcified and 13 lipid-rich non-calcified) from postmortem human coronary arteries were scanned with PCCT [155]. This technique provided distinct visualization and differentiation of normal arterial walls, lipid-rich plaques, calcified areas, and surrounding adipose tissue based on their unique spectral attenuation characteristics and iodine-based contrast agent concentration [155]. A recent study showed no significant differences between PCCT-derived measurements and histological measurements of fibrous cap thickness, fibrous cap area, and lipid-rich necrotic core area [156]. 

In the context of molecular imaging techniques that offer insights into the biological activity within plaques, including inflammation and neovascularization, PCCT has shown promising results. Experiments conducted on phantoms and animals have illustrated the potential of utilizing PCCT with K-edge imaging and gold nanoparticles as an effective strategy for concurrently assessing lumen stenosis, plaque composition, vulnerability, and macrophage detection [157].

In studies using phantoms and Apolipoprotein E knockout mouse models of atherosclerosis, Cormode et al. demonstrated that PCCT could effectively discriminate between gold-based contrast agents, iodinated contrast agents, tissue, and calcium-rich matter [158]. This confirmed PCCT’s ability to detect macrophages in atherosclerosis while simultaneously visualizing vascular structures and calcified tissue. Si-Mohamed and colleagues conducted imaging on both atherosclerotic and control New Zealand white rabbits before, as well as two days after, administering an injection of gold nanoparticles [157]. The results of their study demonstrated that PCCT exhibited a significantly stronger association between gold concentration and macrophage area compared to conventional CT, with correlation coefficients of 0.82 and 0.41, respectively. Furthermore, the study highlighted that only PCCT employing gold K-edge imaging was able to successfully distinguish between the enhancement of the inner lumen, which was achieved using an iodinated contrast material, and the enhancement of the vessel wall, caused by the gold nanoparticles [157].

**Figure 6:**
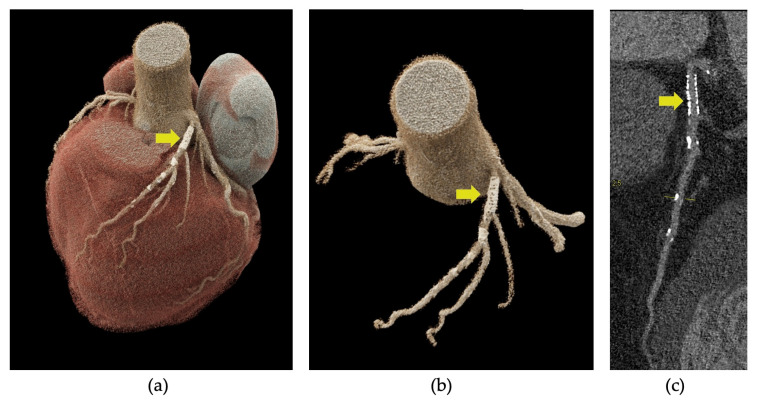
Photon-counting detector CT angiography (PCD-CTA) of the heart showing coronary stent implantation in the proximal left anterior descending artery (LAD) (yellow arrow in **a**–**c**). Three-dimensional cinematic rendering of the heart, showing the stent in the LAD (**a**), three-dimensional rendering of the ascending aorta and left coronary artery and the stent in proximal LAD (**b**), curved multiplanar reformation showing the absence of in-stent stenosis (**c**).

PCCT also enhanced the visualization of the stent lumen, reduced image noise, minimized blooming artifacts, and improved overall image quality, as shown in Figure 6. In their research, Symons et al. discovered that high-resolution PCCT, with a voxel size of 0.25 mm, provided superior visibility of the coronary stent lumen compared to both standard-resolution PCCT, with a 0.5 mm voxel size, and conventional dual-energy CT [159]. This conclusion was further corroborated by a study conducted by Petritsch et al., which demonstrated that PCCT, when operated in ultra-high-resolution mode, delivered the best visibility of the in-stent lumen while scanning a body phantom containing 28 different coronary stents [160].

Even in myocardial tissue characterization, preliminary studies have shown that PCCT could play a significant role. Mergen et al. demonstrated in vivo with 30 patients suffering from severe aortic stenosis that late enhancement scan acquisition with PCCT is feasible at a low radiation dose [161]. The extracellular volume (ECV) measurements derived from both dual-energy and single-energy scans exhibited a high correlation (r = 0.87, *p* < 0.001) with a negligible mean error of 0.9%. Another in vivo study confirmed this strong correlation (r = 0.91, *p* < 0.001) in 29 patients, where dual-energy PCCT provided the advantage of a significantly lower radiation dose (40%) [161]. Importantly, both dual-energy and single-energy PCCT techniques demonstrated strong correlation and good reliability for ECV quantification compared to CMR.

## 11. Cardiac Magnetic Resonance Imaging in Cardiovascular Prevention

Because of limited availability, high costs, and lengthy execution, CMR is rarely used for cardiovascular prevention. However, its significant potential makes it valuable in specific contexts [162]. 

While its role in SCD risk stratification for patients with non-ischemic- and ischemic cardiomyopathy is well-defined [163,164,165], emerging data highlights the potential role of stress cardiovascular CMR and evaluation of chemotherapy toxicity prevention and EAT. 

Stress cardiovascular CMR perfusion imaging is a promising modality for evaluating CAD due to its high spatial resolution and lack of radiation exposure. The semi-quantitative and quantitative analyses of CMR perfusion depend on signal-intensity curves produced during the first pass of gadolinium contrast [166]. Despite the introduction of various semi-quantitative and quantitative modalities, their diagnostic performance varies significantly among studies, and standardized protocols are lacking [167]. 

Even in the field of chemotherapy toxicity prevention, CMR plays an important role as it can early detect alterations in systolic function or myocardial strain, especially in patients with challenging acoustic windows [168]. Moreover, through tissue characterization, CMR is capable of identifying potential myocardial edema and inflammation that may arise after the initiation of oncological therapy [169], such as myocarditis due to the use of immune checkpoint inhibitors [170].

**Figure 7:**
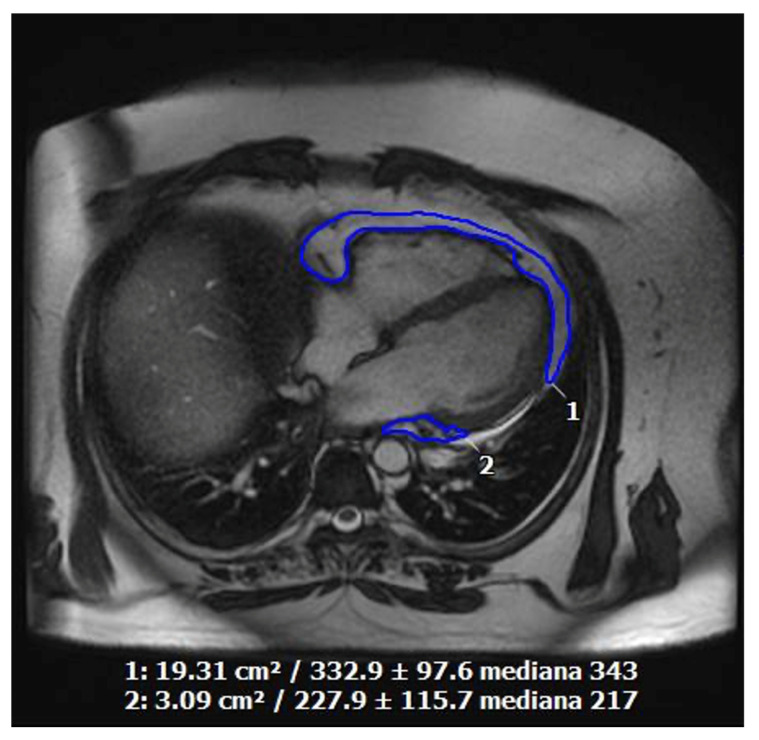
Quantification of epicardial fat by cardiac magnetic resonance imaging in a 55-year-old obese, smoking, dyslipidemic male patient with poor echocardiographic acoustic window and symptomatic for exertional angina.

CMR has also emerged as an effective method for measuring adipose tissue, offering a safe, non-invasive alternative to traditional methods like water immersion or radiation exposure. Its high resolution and clarity enable detailed visualization of adipose tissue, aiding in the identification of abnormalities or changes in tissue distribution indicative of health issues [171]. Balanced steady-state free precession sequences (SSFP) in CMR imaging are particularly adept at distinguishing adipose tissue from blood and muscle, providing the clearest images of EAT distribution to date. EAT extends beyond a superficial layer of fat on the heart’s surface, infiltrating various cardiac structures (Figure 7). Detailed imaging of these extensions requires a significant amount of EAT, as smaller amounts may fall below the spatial resolution limits of current non-invasive techniques [172]. EAT assessment through CMR has shown its value in specific clinical scenarios. For instance, patients with Takotsubo cardiomyopathy exhibit higher epicardial fat volumes compared to healthy individuals of similar body mass index [171]. The amount of EAT correlates with markers of myocardial inflammation and early signs of contractile dysfunction, as seen on CMR [171]. Similarly, research by Yuan W. et al. demonstrated that in patients with Duchenne muscular dystrophy, the development of late gadolinium enhancement-based myocardial fibrosis is associated with an increased EAT volume. Incorporating EAT volumes into a predictive nomogram enhanced the accuracy of identifying myocardial fibrosis in these patients [173].

Stronger data about the role of CMR in EAT evaluation in cardiovascular prevention are needed.

## 12. Artificial Intelligence in Cardiovascular Imaging for Risk Assessment

Artificial intelligence (AI) is a branch of computer science focused on developing human cognitive processes. Machine learning (ML), the foundation of AI, employs data-based models to facilitate decision-making and algorithms programmed to learn, perform tasks, or solve specific problems automatically [174]. Deep learning (DL), a specific method of ML algorithms, mimics the learning process of the human brain by using artificial neural networks, enabling computer systems to interpret, construct, and comprehend complex data structures and hierarchies [175]. 

The vast amount of real-world data available on CVD risk factors and prevention has created an opportunity for the application of artificial intelligence (AI) to revolutionize personalized medicine and tailored primary or secondary prevention strategies [176,177]. AI techniques can be seamlessly integrated into imaging tools commonly used in the evaluation of CVD, such as CT, echocardiography, and CMR [178]. For instance, in echocardiography, AI-learned patterns can automatically calculate LVEF, providing clinicians with valuable information about cardiac function without the need for manual calculations. Additionally, AI can automate the quantification of left and right ventricle volumes and atrial size and function from both 2D and 3D acquisitions [179,180,181].

Interestingly, DL technologies have been successfully applied to diagnose regional wall motion abnormalities [182] and automate the measurement of global longitudinal strain in patients with a history of AMI [183]. 

Future applications of AI in TTE are vast, particularly in the field of valvular heart disease (VHD) and cardiomyopathy. As regards VHD, a support vector machine emerged as the most accurate in determining the severity of mitral regurgitation, while another study found that ML calculations were closely correlated with human measurements of aortic stenosis severity [184,185]. Furthermore, in an interesting study conducted by Yang et al., a DL algorithm demonstrated high accuracy in identifying VHD such as mitral regurgitation, mitral stenosis, and aortic stenosis [186]. 

The right algorithms can aid in differential diagnosis in complex situations, such as differentiating between hypertrophic cardiomyopathy and athlete’s heart [187], between restrictive cardiomyopathy and constrictive pericarditis [188], or even between myocardial infarction and Takotsubo syndrome [189]. 

Looking to the future, the data from the initial experiences of applying ML to stress echocardiography appear very promising [190]. In this way, the Prospective Randomized Controlled Trial Evaluating the Use of Artificial Intelligence in Stress Echocardiography trial will assess the impact of an AI medical diagnostic aid on the standard care pathway of patients with suspected CAD being investigated with stress echocardiography [191]. 

AI also has shown promise in enhancing the evaluation of CAD using CT imaging. Automated systems have been developed to rapidly and accurately assess stenosis, atherosclerosis, and vessel morphology on CCTA scans, reducing the time and effort required for interpretation. Due to the risk of image deterioration and motion artifacts in CT, the use of DL algorithms has enabled cleaner processing of acquired images. Previous studies have demonstrated that AI-enhanced image processing results in higher image quality and a level of accuracy in coronary artery stenosis that closely matches invasive stratification methods, including intravascular ultrasound [189].

Notably, artificial intelligence–guided analysis of quantitative computed tomography (AI-QCT) has demonstrated high diagnostic accuracy, enabling rapid and objective quantitative assessment of adverse plaque characteristics such as total plaque volume, plaque morphology, vessel volume, vessel involvement, and stenosis to guide risk stratification and therapy [192,193,194]. A recent post hoc analysis of the CREDENCE (Computed Tomographic Evaluation of Atherosclerotic Determinants of Myocardial Ischemia) study (n = 612) [195] and the PACIFIC-1 (Prospective Comparison of Cardiac PET/CT, SPECT/CT Perfusion Imaging and CT Coronary Angiography With Invasive Coronary Angiography) study (n = 208) [196], by Nick S. Nurmohamed et al. [193], examined an AI-QCT model for the diagnosis of coronary ischemia. This model, known as AI-QCTISCHEMIA, integrated atherosclerosis and vascular morphology measures to evaluate its prognostic utility for MACE. The diagnostic performance by the area under the receiver-operating characteristics curve on a per-patient level was 0.80 (95% CI: 0.75–0.85) for AI-QCTISCHEMIA. Moreover, after adjusting for clinical risk factors and coronary CTA-determined obstructive stenosis, a positive AI-QCTISCHEMIA test was associated with an HR of 7.6 (95% CI: 1.2–47.0; *p* = 0.030) for MACE.

Studies have also confirmed that DL algorithms can provide reliable measurements of atherosclerotic burden, plaque thickness, plaque volume, and stenosis severity, significantly impacting the estimation of prognosis of AMI [197]. Beyond imaging techniques, AI has been utilized to predict cardiovascular events based on other data sources, such as the quantification of CAC on CT scans. ML algorithms for identifying CAC have shown very promising results, with a strong correlation with measurements made by human experts [198]. These algorithms have also proven to be strong predictors of cardiovascular events, independent of traditional risk factors [199]. Similarly, AI algorithms have been used to quantify EAT volume and predict major cardiovascular events in asymptomatic patients, demonstrating the potential for AI to provide prognostic information without the need for additional imaging techniques [200].

The widespread use of CMR has sparked significant interest in applying AI to enhance the accuracy, reproducibility, and precision of examinations [201].

Several studies have demonstrated the success of ML-based algorithms in automating and accelerating the acquisition process [202,203]. For instance, generative adversarial networks enable the creation of cine-like CMR images from real-time sequences, which greatly benefits patients who have difficulty holding their breath or those with arrhythmias, thereby enhancing image quality [204].

Moreover, automated ML analysis provides more precise and standardized evaluations, essential for consistent cardiac function assessment [205,206,207]. AI also shows great potential in tissue characterization parameters, such as late gadolinium enhancement and T1–T2 mapping, which are critical for both diagnostic and prognostic models [164,208,209,210]. This application of AI in CMR is particularly important for better stratification and prognosis in patients with HF or cardiomyopathies, or those undergoing invasive treatments [211,212].

While the potential benefits of AI in cardiovascular medicine are vast, it is important to recognize the limitations and ethical considerations associated with AI technologies. Transparency and fairness in AI algorithms are critical to ensure that decisions made by these systems are ethical and trustworthy [213].

Overall, the application of AI in CVD risk prediction and prevention represents a significant advancement in the field of cardiovascular medicine. With the rapid growth of AI technologies and the increasing availability of real-world data, the use of AI in cardiovascular medicine has the potential to transform the way healthcare professionals predict and prevent cardiovascular events [214]. Despite the challenges and uncertainties that come with the integration of AI in medicine, the potential benefits of AI in enhancing personalized medicine and improving patient outcomes are substantial, giving hope for a future where AI plays a primary role in cardiovascular risk assessment and prevention strategies.

## 13. Conclusions

In conclusion, cardiovascular imaging could be a pivotal tool in assessing cardiovascular risk across a wide range of patients. Emerging imaging modalities present the opportunity to identify novel risk markers, which can further enhance our understanding of cardiovascular risk, identify evidence of early-stage disease, and aid in targeted interventions to prevent cardiovascular events. Extensive studies are needed to evaluate the cost-effectiveness of using such methods on a large scale. 

The integration of AI into cardiovascular imaging holds great promise for the future of cardiovascular risk assessment and prevention. AI has the potential to automate and streamline the interpretation of imaging studies, allowing for faster and more accurate risk stratification. This technology can aid in the identification of subtle abnormalities on imaging studies that may not be apparent to the human eye, further improving risk assessment and patient care.

Looking ahead, continued research and advancements in cardiovascular imaging will undoubtedly improve our ability to assess and manage cardiovascular risk, ultimately leading to better outcomes for patients.

## Figures and Tables

**Table 1:** Key studies on prognostic role of high-risk plaque characteristics investigated by coronary computed tomography angiography.

	Setting	Study Design	Follow-Up	Patients Number	Plaque Characteristics	ACS or MACE Prediction Rates
Feuchtner et al. [113]	Suspected CAD	Observational	7.8 years	1469	LAP, PR, NRS, SC	MACE group showed decreased LAP (35.2 HU ± 32 vs. 108.8 HU ± 53; *p* < 0.001), increased NRS presence (63.4% vs. 28%; *p* < 0.001)
Ferencik et al. [114]	Stable CAD	RCT	25 months	4415	LAP, PR, NRS	HRP was associated with higher MACE rate (6.4% vs. 2.4%; HR: 2.73; 95% CI, 1.89–3.93)
Chang et al. [115]	Stable CAD and ACS	Observational	3.4 ± 2.1 years	468	LAP, PR, SC	ACS events were predicted by HRP presence (HR: 1.59; 95% CI: 1.22 to 2.08), SC presence (HR:1.54; 95% CI:1.17–2.04), LAP presence (HR: 1.38; 95% CI: 1.05–1.81)
Williams et al. [116]	Stable CAD	RCT, post-hoc analysis	4.7 years (IQR 4.0–5.7)	1769	LAP, PR, NRS, SC	MACE were associated with increased HRP (4.1% vs. non-HRP 1.4%; *p* < 0.001) Obstructive CAD was associated with increased HRP (4.9% vs. non-HRP 2.4%; *p* = 0.036)
Senoner et al. [117]	Suspected CAD	Observational	10.55 ± 1.98 years	1430	LAP, PR, NRS, SC	MACE were predicted by LAP < 60 HU (HR: 4.00, 95%CI: 1.52–10.52, *p* = 0.005), NRS (HR 4.11, 95% CI: 1.77–9.52, *p* = 0.001) SC and PR not significant
Taron et al. [118]	Stable CAD	Observational	26 months	2890	LAP, PR, NRS	≥2 HRP (vs. <2 HRP) features predicted MACE (HR: 2.25, 95% CI: 1.01–5.01, *p* = 0.04)
Yang et al. [119]	Stable CAD	Observational	2.9 years	335	LAP, PR	MACE were predicted by HRP (HR: 2.70; 95% CI: 1.10–6.50; *p* = 0.02)

Abbreviations: ACS—acute coronary syndrome; CAD—coronary artery disease; CI—confidence interval; HR—hazard ratio; HRP—high-risk plaque; HU—Hounsfield units; LAP—low-attenuation plaque; MACE—major adverse cardiovascular events; MI—myocardial infarction; NRS—napkin-ring sign; PR—positive remodeling; SC—spotty calcification.

## Data Availability

All data generated or analyzed during this study are included in this published article. Further inquiries should be directed to the corresponding author.
